# Lightweight Semantic-Guided Neural Networks Based on Single Head Attention for Action Recognition

**DOI:** 10.3390/s22239249

**Published:** 2022-11-28

**Authors:** Seon-Bin Kim, Chanhyuk Jung, Byeong-Il Kim, Byoung Chul Ko

**Affiliations:** Department of Computer Engineering, Keimyung University, Daegu 42601, Republic of Korea

**Keywords:** action recognition, semantic-guided neural networks, single head attention, graph convolutional networks, skeletal structure

## Abstract

Skeleton-based action recognition can achieve a relatively high performance by transforming the human skeleton structure in an image into a graph and applying action recognition based on structural changes in the body. Among the many graph convolutional network (GCN) approaches used in skeleton-based action recognition, semantic-guided neural networks (SGNs) are fast action recognition algorithms that hierarchically learn spatial and temporal features by applying a GCN. However, because an SGN focuses on global feature learning rather than local feature learning owing to the structural characteristics, there is a limit to an action recognition in which the dependency between neighbouring nodes is important. To solve these problems and simultaneously achieve a real-time action recognition in low-end devices, in this study, a single head attention (SHA) that can overcome the limitations of an SGN is proposed, and a new SGN-SHA model that combines SHA with an SGN is presented. In experiments on various action recognition benchmark datasets, the proposed SGN-SHA model significantly reduced the computational complexity while exhibiting a performance similar to that of an existing SGN and other state-of-the-art methods.

## 1. Introduction

In the fields of computer vision and machine learning, human behaviour recognition aims to recognise human intention and provide appropriate services that match this intention. Therefore, behaviour recognition is being applied to various fields, such as human–machine interfaces (HMI), surveillance systems, games, augmented reality, and virtual reality.

Human behaviours can be largely divided into independent behaviours and interactions. An independent behaviour means that different body parts move continuously, and an interaction means that two or more objects provide and receive actions [1]. Independent behaviours can be further divided into actions and activities. An action refers to a behaviour that occurs during a relatively short period of 20–30 frames. Example actions include ‘running’, ‘walking’, and ‘bending’. An activity is a collection of actions in which actions occur continuously for a relatively long time and various actions are simultaneously combined. Example activities include ‘opening a car door and driving’ and ‘taking a smartphone out of a pocket and making a call’. By contrast, an interaction refers to an action exchanged between a human being and an object (human or object), such as ‘shaking hands’, ‘fighting’ and ‘hugging’.

The purpose of this study is to recognise real-time human actions occurring within a short time. We therefore focus on action recognition rather than activity recognition.

Action recognition includes sensor- and vision-based methods using a camera. The sensor-based method incurs an additional cost of purchasing the sensors and a reluctance to wear the sensors. Therefore, vision-based action recognition has primarily been studied. Vision-based action recognition can be further divided into a method using only the RGB of video frames, a method using only the human skeleton (pose) of a video, and a method using a video frame and a skeleton together. As an advantage of video-frame-based action recognition [1,2], the model structure is simple because only visual information is used. However, the performance may degrade because it reacts sensitively to background noise (e.g., camera angles, body sizes, and complex backgrounds) or changes in illuminance. Conversely, skeletal-based action recognition [3,4] involves structural changes in the body by converting the skeletal structure into features. This approach can achieve a high performance because it is less affected by various real-world noises. However, it has the disadvantage of requiring a separate model for extracting the skeleton data. When RGB video frames and skeletons are used together, ambiguous actions (e.g., ‘playing tennis’ and ‘playing badminton’) with the same pose can be distinguished using RGBe information. However, it is difficult to combine two sets of cross-modal information, and when individual features are applied to each recognition module, there is a limit to real-time action recognition. Therefore, this study introduces a skeleton-based action recognition algorithm with a fast learning speed that can recognise actions in real time.

In skeleton-based action recognition, a method of extracting spatiotemporal features from skeleton sequences and combining them with a graph convolution network (GCN) has been widely used. Among them, semantic-guided neural networks (SGNs) [5] are fast and promising action recognition algorithms that hierarchically learn the spatial and temporal features using a GCN. However, because an SGN focuses on global feature learning rather than local feature learning owing to the structural characteristics, there is a limit to action recognition in which the dependency between neighbouring nodes is important. In addition, although an SGN is a lightweight model, it still has a problem in that it has a considerable computational complexity for real-time action recognition. Therefore, in this study, although an SGN was used as a base network for action recognition, the graph structure was replaced with a single-head attention (SHA) structure to enable real-time action recognition in low-end devices. In this way, the action recognition performance can be improved, and the number of parameters and the computational complexity can be dramatically reduced.

The contributions of this paper are as follows: 

(1) By converting the joint-level module for spatial feature extraction of the skeleton joints into a single SHA instead of a GCN, we can create spatial features emphasizing important joints.

(2) To embed the spatial characteristics of the joints, in contrast to a GCN, an SHA composed of a query, key, and value can emphasise the characteristics between joints.

(3) The proposed joint-level module reduces the loss of the input features and emphasizes the embedded features by combining the residual network with the SHA output.

(4) Through various experiments, we proved that the proposed SGN-SHA model can reduce the number of parameters by half, and the number of operations by 2.6-fold compared to the base SGN without lowering the accuracy compared to other models.

The remainder of this paper is organised as follows. In Section 2, we present a review of related studies on the three different approaches to action recognition. Section 3 provides details of the proposed SGN-SHA method. Section 4 provides a comprehensive evaluation of the proposed method, based on various experiments conducted. Finally, some concluding remarks are presented in Section 5.

## 2. Related Studies

As described above, studies related to computer-vision-based action recognition can be divided into three categories according to the input data types: an approach using the RGB of the video frames, an approach using skeleton sequences, and an approach using the RGB and skeleton sequences together. This section introduces details of previous related studies for each category.

### 2.1. RGB of Video Frame-Based Action Recognition 

Baradel et al. [2] presented a human action recognition method that does not require joint poses during testing, and modelled the action using two attention processes. These two methods allow the recognition of human action in unstructured spatio-temporal collections of glimpses through distributed iterative tracking/recognition and a soft assignment between the glimpse points and trackers.

Although most RGB video frame-based action recognition methods use spatio-temporal information, the time information cannot be obtained under a static state. Girish et al. [6] decomposed spatial actions in still images into smaller semantic components and proposed a CNN model that can understand the importance of each component. Wang et al. [7] proposed an action recognition method based on the spatio-temporal, channel, and motion excitation modules in the RGB of the video frames. To achieve a high fidelity, this algorithm uses (1) a one-channel 3D convolution to characterise the spatiotemporal representations, (2) explicit modelling of the interdependencies between channels in terms of time, and (3) a stimulation of motion-sensitive channels. Feichtenhofer [8] proposed an extended 3D architecture network that progressively extends a small 2D image classification architecture along multiple network axes in space, time, width, and depth. This method proved that the accuracy of complex actions can be increased through the use of a simple stepwise network expansion instead of a complex network.

Unlike existing methods that partially recognise an action by dividing the video frames, Liu et al. [9] proposed an action recognition approach using full video frames. For computational efficiency, this method first clusters all frames along the time dimension based on the similarity associated with the classification task, and then aggregates the frames in the cluster into fewer representations. Therefore, this method enables a fast action recognition by using temporally localised clustering with a fast Hamming distance in the feature space. Wang et al. [10] proposed a transformer-based encoder-decoder framework called OadTR to solve the spatiotemporal problem of action recognition. Encoders associated with task tokens in video frames aim to capture global interactions and relationships between historical observations. The decoder extracts auxiliary information by aggregating the expected future clip representations. Thus, the OadTR can recognise current actions by encoding past information and simultaneously predicting future contexts.

The RGB of video frame-based action recognition has an advantage in that it utilises the RGB information of the video frame as-is and does not require additional information, such as the skeleton. However, noise in the wild (e.g., the camera angle or an obscured pose) and the complex background generated during image acquisition are the main factors lowering the accuracy of the action recognition.

### 2.2. Skeleton-Based Action Recognition

Yan et al. [4] proposed a spatial-temporal graph convolutional network (ST-GCN), which can improve the performance of an action recognition by automatically learning the spatial and temporal patterns from skeleton sequences extracted from video frames. An ST-GCN has a greater expressive power and generalisation capability for spatiotemporal skeleton features. Thakkar and Narayanan [11] divided a skeleton graph into four subgraphs and proposed an action recognition model using a part-based GCN. They also showed that this model has an improved recognition performance compared to a model using a full skeleton graph. Li et al. [5] proposed an actional-structure GCN that adds actional- and structural-link structures to the ST-GCN for representing richer joint relationships. An action link is composed of an encoder–decoder structure, through which the relationships between joints connected nearby and between joints at a longer distance can be found. However, ST-GCN-based methods have a disadvantage in that they require a large number of operations compared with their accuracy.

To solve this problem, Cheng et al. [12] proposed a shift-graph convolution network. This method consists of a spatio-temporal shift graph operation and a light point-wise convolution instead of a heavy general graph convolution, through which, the action recognition performance is improved. Zhang et al. [1] proposed an efficient SGN. High-level joint semantics (e.g., type of joints) or frame indices (number of frames) have been used to enhance the feature representation capability. Cai et al. [13] proposed an action recognition framework for jointly using a human pose skeleton and lightweight joint-centric information in a dual-stream GCN called a JOLO-GCN. Compared with single-skeleton-based methods, this hybrid approach has been proven to effectively improve the performance while maintaining a low computational and memory overhead. Mazzia et al. [14] proposed an action transformer, which is a simpler yet self-attentional architecture that combines convolutional, recurrent, and attentive layers. This method enables real-time pose-based action recognition using a 2D pose representation and a low-latency solution within small temporal windows, reducing the number of operations required for an action recognition.

Although GCN-based motion recognition shows an excellent performance in skeleton-based action recognition, it has the disadvantage of increasing the computational complexity and the number of parameters, as with other approaches, when a spatio-temporal video sequence becomes excessively long.

### 2.3. Video Frame and Skeleton Based Action Recognition

Zhu et al. [15] detected human bounding boxes in video frames and recognised human actions by fusing RGB with a skeleton. This method adds a branch for human pose estimation and a 2D CNN for pose-based action recognition. This allows the method to benefit from the multitask training of action recognition and pose estimation and the predictive fusion of RGB images and poses. Das et al. [16] proposed a video pose network (VPN). A VPN comprises two key elements: spatial embedding and state networks. Utilising both networks and video pose features, the action recognition framework can better learn the spatio-temporal features. Joze et al. [17] presented a simple multimodal transfer neural network module for utilising the information obtained from various modalities (e.g., RGB, sound, skeleton, and depth). This method fuses multimodal information and utilises squeeze and excitation operations in the network. The action recognition performance is improved by recalibrating the characteristics of each channel of the CNN stream. Davoodikakhki and Yin [18] proposed an algorithm that captures only the human part from the RGB of video frames and combines the extracted skeleton data, allowing the ResNet model to focus solely on human action during the learning process. When combined in this manner, the shortcomings of an action recognition can be overcome. Zhu et al. [19] proposed a multimodal feature fusion network that combines the features of skeleton sequences and RGB frames. A two-step fusion framework is introduced into the network to better explore the correspondence between two contrasting RGB and skeleton features. In this way, complementary information is maintained, while at the same time, the complexity is significantly reduced.

This multimodal feature fusion method can improve the action recognition performance by fusing the skeleton sequence and RGB features of the video frames. However, apart from the network used for extracting the features from the RGB of the video frames, this approach requires a separate network for extracting the skeletons. In addition, this approach combines two heterogeneous features and requires a downstream network for action recognition. Therefore, it sacrifices the efficiency for the high network complexity.

## 3. Proposed Method

In this section, we introduce a light and fast action-recognition algorithm based on a skeleton and a combination of the proposed SHA and SGN. In a base SGN, the input skeleton sequence is divided into joint-level (position, 3D coordinates, and velocity) and frame-level (joint type and frame index) modules, and is used for action recognition. Because it is composed of two heavy modules, considerable computational complexity and model parameters are required. By replacing the joint-level module with an SHA, which requires fewer operations, real-time action recognition in low-end devices is possible while maintaining the level of accuracy.

### 3.1. Overall Architecture of SGN-SHA

**Figure 1:**
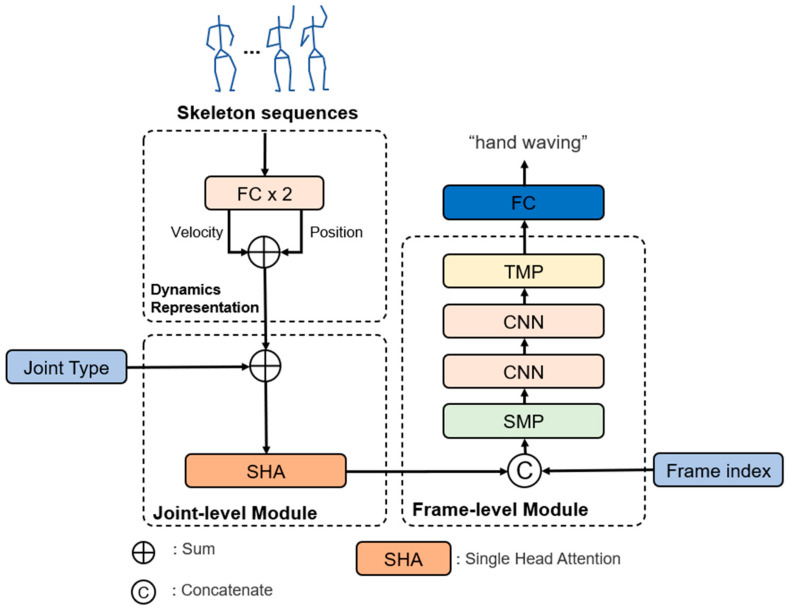
Illustration of the proposed SGN-SHA, which consists of three consecutive modules, i.e., dynamic representation, joint-level, and frame-level modules. The joint-level module can significantly reduce the numbers of operations and parameters by applying the proposed SHA instead of multiple GCNs.

In an SGN [1], the base algorithm of our study, the spatiotemporal information of the skeleton sequence is embedded by arranging three modules sequentially, i.e., dynamic representation (DR), joint-level, and frame-level modules, as shown in Figure 1. The role of the DR is to encode/embed the position and velocity in the same high-dimensional space and fuse them. The joint-level module is used to exploit the correlation of the joints in the same frame. Finally, a frame-level module is used to exploit the correlation between frames.

**Figure 2:**
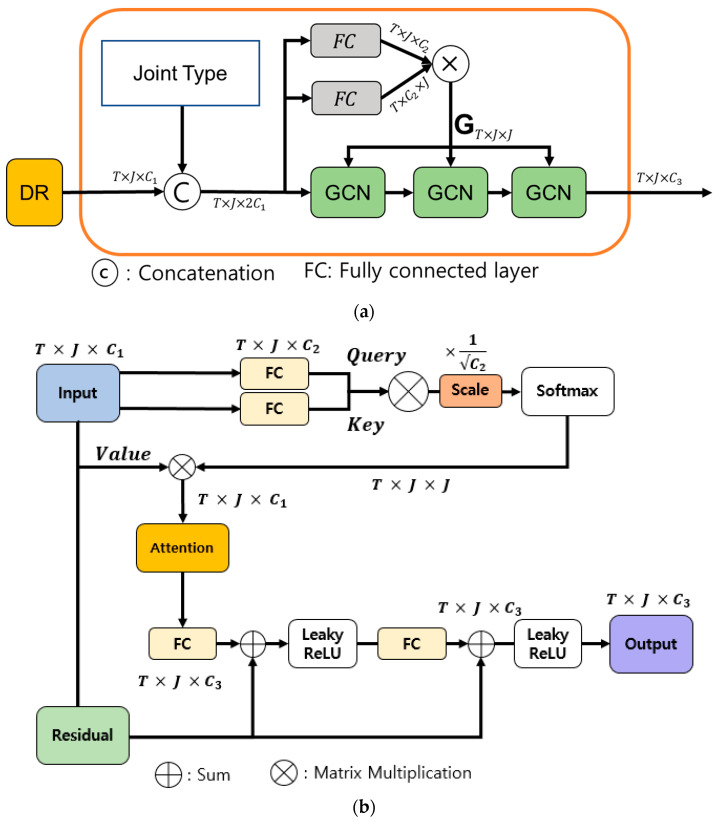
Illustration of the joint-level module of (**a**) original SGN based on a GCN and (**b**) the proposed SHA. (**a**) The output of the DR and the joint type vector are concatenated to create a joint-level feature through three consecutive GCNs. (**b**) A joint-level feature is created that emphasizes important joints through four one-layer FCs and a residual network.

Although the base SGN shows an excellent action recognition accuracy with a relatively low number of operations compared to other state-of-the-art (SoTA) methods, there is a problem in that it requires unnecessary operations and parameters owing to the structural problem of a GCN in the joint-level module. The embedding feature of the DR module shown in Figure 1 must be converted into a graph form in the joint-level module (see Figure 2a). Therefore, the node features of each joint may be incorrectly matched during the graph transformation process. In addition, because the adjacency matrix must be maintained for each frame and the node features must be applied with the adjacency matrix to three consecutive GCN layers, resulting in an increase in the amount of memory and number of operations. Therefore, in this study, a new joint-level module based on SHA (see Figure 2b) is proposed to maintain the accuracy while significantly reducing the numbers of operations and parameters of an SGN.

Figure 1 shows our proposed SGN-SHA, which was developed according to the following sequential procedure:

### 3.2. Dynamic Representation Module

First, we denote all joints as a set $S=\left\{X_{t}^{k}|t=1,2,\ldots ,T;k=1,2,\ldots ,J\right\}$ for the skeleton sequence, where $X_{t}^{k}$ denotes a joint of type *k* at time *t*, and a joint is represented as a 3D position (x,y,z). In addition, *T* represents the number of frames in the skeleton sequence, and *J* represents the total number of joints in the human body in a single frame.

In the DR module, the joint position and velocity information are transformed separately using a fully connected (FC) layer. The joint velocity uses the difference in the distance between joint $X_{t}^{k}\;$ of the *T* frame and the joint of the *T-1* frame. The output features are then concatenated as a feature representation with a dimension of $T\times J\times C_{1}$, where $C_{1}$ is the dimension of the joint representation. The output of the DR module is combined with the joint type (e.g., head and foot) and input into the SHA to model the correlation of joints in the same frame.

### 3.3. Single Head Attention of Joint-Level Module

The purpose of the joint-level module is to exploit the correlations between joints in the same frame. The joint-level module of the base SGN consisted of two FCs and three consecutive GCNs, as shown in Figure 2a. Therefore, the learning parameters and adjacency matrices for an individual GCN are required, and a large number of operations is required for the GCN operation. The proposed SHA, as shown in Figure 2b, which is inspired by the attention mechanism of the transformer [20], can represent a new dimension of the output that reflects not only the global features of the skeleton but also the local features that consider the dependencies between adjacent joints.

The attention head, the core idea of a transformer, calculates the attention between input tokens (features) using three matrices: query (Q), key (K), and value (V). Transformers use multi-head attention (MHA) such that *p* heads can learn a different attention from the input and combine them to obtain a strong attention representation [20]. However, it has been proven that only some of the multiheads affect the attention performance of the transformer, and some heads pay attention to unnecessary parts [21]. Therefore, instead of the complex joint-level modules of the base SGN model, we replaced the transformer MHA with SHA. Even if we use only SHA instead of MHA, we can create spatial features emphasising important joints and secure a smaller number of parameters and a lower computational complexity than the base SGN.

First, the input vector $I\in {\mathbb{R}}^{T\times J\times C_{1}}$ summed with the output of the DR and joint type is embedded as Q and K through a separate FC.

(1) $$Q={\mathrm{FC}}^{q}\left(I\right)\;\in {\mathbb{R}}^{T\times J\times C_{2}}\;$$

(2) $$K={\mathrm{FC}}^{k}\left(I\right)\;\in {\mathbb{R}}^{T\times J\times C_{2}}$$

The attention is calculated using the scaled dot product of Q and transposed K. The softmax operation result is again produced with V to generate the final attention.

(3) $$\mathrm{Attention}\;\left(Q,K\right)=\mathrm{softmax}\left(\frac{{\mathrm{QK}}^{T}}{\sqrt{\;C\prime }}\right)V\in {\mathbb{R}}^{T\times J\times J}\;$$

Unlike a pure transformer, the input vector is also replaced with $V\in {\mathbb{R}}^{T\times J\times C_{1}}$ and multiplied by the attention as-is without going through a separate FC. The attention is entered into the FC and transformed into dimensions of $T\times J\times C_{3}\;$ to create new features that consider such attention.

The FC output conducts a sum operation with the same-sized output of a residual network composed of a single layer. Residual connections between layers are used to reduce the loss of the input features and emphasise the embedded features. The final $T\times J\times C_{3}\;$ dimensional joint feature representation is obtained by sequentially applying the sum operation with the leaky ReLU, FC, and residual networks. The final output of the joint-level module is fed to the frame-level module to exploit the correlations across frames.

Through the proposed SHA, we prove in Section 4 that if the FC for the Q and K matrices is trained to emphasise the important joints, the attention of the important joints can then be obtained using only the SHA.

### 3.4. Frame-Level Module

The frame index is concatenated with the output features $T\times J\times C_{3}\;$ of the joint-level module and input into the spatial max-pooling, which compresses the spatial dimension. It is then input into the temporal max pooling (TMP) which compresses the temporal dimension after passing through two CNNs. The TMP aggregates the information of all frames and obtains the sequence-level feature representation with $C_{3}\;$ dimensions. The $C_{out}$ feature vector applies the final classification through the last FC using softmax.

## 4. Experimental Results

In this section, we introduce datasets to be used for human action recognition experiments, and through comparative experiments, we prove that the proposed method is promising compared to other state-of-the-art (SoTA) methods on various datasets.

### 4.1. Dataset

We measured the accuracy of the skeletal dataset obtained in various environments to prove that the proposed method exhibits a highly robust performance for various actions.

**NTU-RGB+D 60 (NTU60)** [22]: The NTU-RGB+D 60 dataset is a depth- and RGB-based action recognition benchmark for human action recognition, containing more than 56,000 video samples and more than 4 million frames collected from 40 individual subjects. It provides four types of action information, i.e., a depth map, 3D skeletal joint positions, RGB frames, and infrared sequences. This dataset contains 60 different action classes, including 40 daily actions, 11 mutual actions, and 9 health-related actions. It uses two evaluation protocols, i.e., cross-subject (XSub) and cross-view (XView) protocols.

**NTU-RGB+D 120 (NTU120)** [23]: NTU RGB+D 120 is an extension of NTU 60, collected from 106 individual subjects, and contains over 114,000 video samples and over 8 million frames. This dataset contains 120 different action classes that extend beyond NTU60. It has two evaluation protocols, i.e., XSub and cross-setup (XSet) protocols.

**NW-UCLA DB** [24]: The NW-UCLA dataset contains RGB, depth, and human skeletal data captured simultaneously using three Kinect cameras. The skeleton contains 20 joints and 19 bone connections. This dataset contains 10 action categories of 10 people, including ‘pick up with one hand’, ‘pick up with two hands’, ‘drop trash’, ‘walk around’, ‘sit down’, and ‘stand up’.

### 4.2. Implementation Details

All experiments were conducted on an RTX 2080Ti GPU with a Pytorch framework. Training was applied equally for all datasets for up to 120 epochs. The batch size of the dataset was also set to 64. All FCs consisted of a single layer, and batch normalisation, Leaky ReLU, and an Adam optimiser were used with a weight decay of 0.0005. The initial learning rate was 0.001 for the NTU dataset and 0.01 for the UCLA dataset, and LambdaLA (lr_lambda = lambda, epoch = 0.93 ** epoch) provided by Pytorch was used as the learning rate scheduler. The cross-entropy loss was used for classification training.

### 4.3. Performance Comparison on NTU60

In this section, we use the NTU60 dataset, which is widely applied in action recognition, to prove that when SHA is combined with an SGN, the model can be lightweight while maintaining the action recognition performance. Through an experiment, the performance was compared using two evaluation protocols of NTU60, i.e., XSub and XView.

The SoTA methods used in the comparative experiment were (1) the base SGN [1], (2) ST-GCN [4], (3) a shift graph convolutional network (Shift-GCN) [12], (4) an Info-GCN for representation learning for the human skeleton [25], (5) disentangling and unifying graph convolutions (MS-G3D) [26], (6) a decoupled spatial-temporal attention network (DSTA-Net) [27], (7) 3S-aimCLR based on contrastive learning from extremely augmented skeleton sequences [28], (8) rich activated GCN (RA-GCNv2) [29], and (9) the proposed SGN-SHA.

As can be seen from the experimental results in Table 1, Info-GCN achieved the highest accuracy for XSub and XView of NTU60. These results are 5.5% higher than those of the proposed SGN-SHA in terms of XSub and 4.5% in terms of XView. However, because Info-GCN requires 4.8-times more parameters and 29.5-times more computational complexity than SGN-SHA, it can be seen that SGN-SHA is more feasible in terms of real-time processing. Although the difference in accuracy between SGN-SHA and other methods, except Info-GCN, is not large, SGN-SHA is significantly superior in terms of the number of parameters and computational complexity. In particular, compared to the base network SGN, although SGN-SHA has a slightly lower accuracy than the SGN by 1.5% in XSub and 1.9% in XView, it outperforms the SGN by approximately 2.15 in terms of the number of parameters and 2.58 in terms of the computational complexity. Considering that there was little difference from the SoTA methods in terms of the accuracy, as shown in Table 1, the large reduction in the computational complexity of the proposed SGN-SHA is significant because it enables real-time action recognition in low-end devices.

### 4.4. Performance Comparison on NTU120

In addition, experiments were conducted using the NTU120 dataset, which was divided into two evaluation protocols: XSub and XSet. As shown in Table 2, SGN-SHA also showed the smallest number of parameters and considerably fewer operations. Because the Info-GCN used a complex network, it still showed the highest recognition rate even when the number of classes increased from 60 to 120. Although the proposed SGN-SHA achieved a lower accuracy than Info-GCN, compared to 3s-AimCLR, which is an SoTA method, it is 1.6% lower in terms of XSub but 6.2% higher in terms of XSet. In particular, compared with the base SGN, XSub was slightly lower at 0.7%, whereas XSet was rather high at 5.6%. From the results in Table 2, we can confirm that even if the number of classes increases, the performance of SGN-SHA does not significantly decrease, and it still has fewer parameters and a lower computation complexity suitable for real-time processing.

### 4.5. Performance Comparison for the Number of Attention Heads

In this experiment, we compared the performance according to the number of attention heads. To this end, we measured accuracy, number of parameters, and computational complexity by increasing the number of attention heads for the NTU60-XView dataset. As shown in the experimental results in Table 3, the accuracy increased as the number of attention heads increased. When the attention head is 2, the accuracy increases by 1.7%, but on the contrary, the number of parameters increases by about 2.97 times and the computational complexity increases by 4.68 times. In terms of accuracy, it can be seen that as the attention head increases, the accuracy does not increase significantly and converges. However, the number of parameters and computational complexity increase exponentially. In particular, computation complexity, an important factor in low-end embedded systems, increased up to 47 times when the number of heads increased from 1 to 5. From the experiments, we can confirm that the proposed SHA is a reasonable algorithm for action recognition in low-end systems by requiring low computational complexity while maintaining accuracy.

### 4.6. Performance Comparison on NW-UCLA Datasets

**Figure 3:**
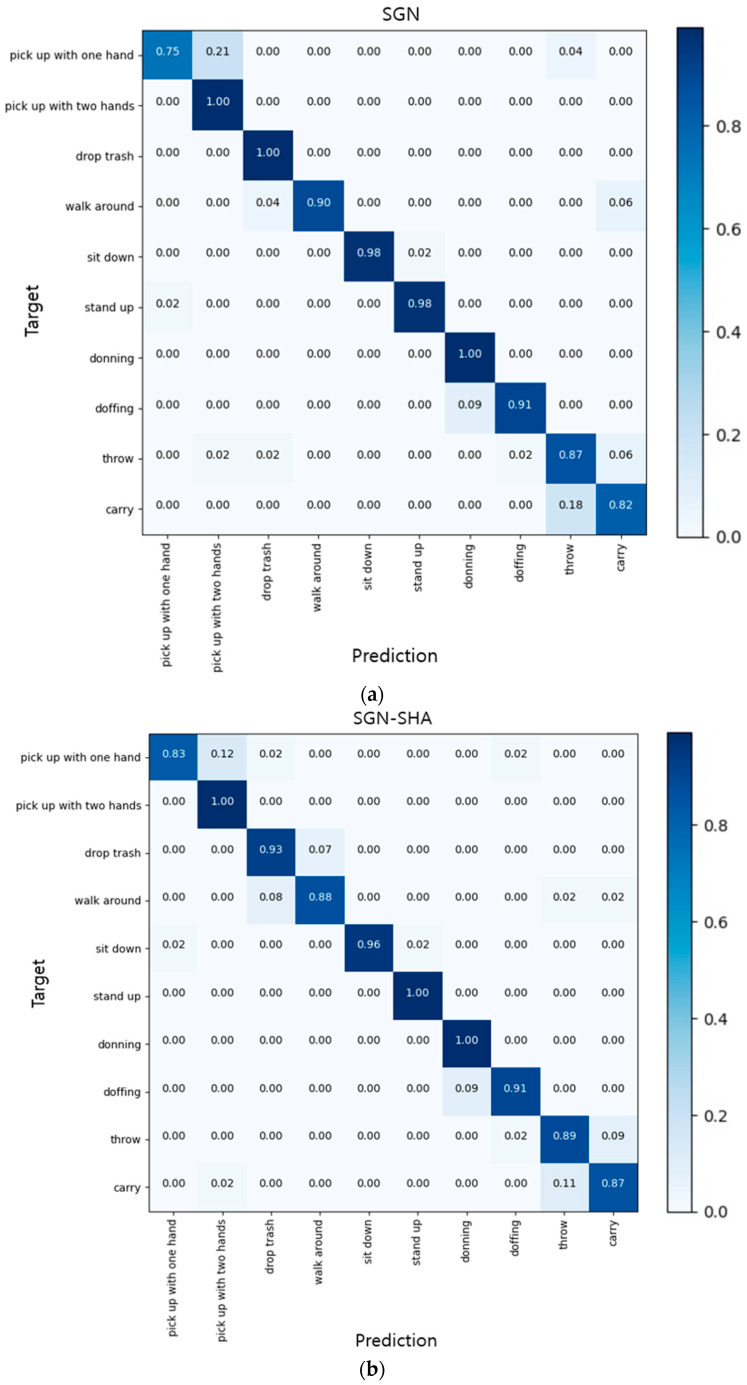
Comparison of confusion matrix accuracy of (**a**) SGN and (**b**) SGN-SHA for detailed actions in NW-UCLA dataset.

The NW-UCLA dataset consists of 10 action classes, and the overall action accuracy is 92.5% for the basic SGN and 92.6% for the SGN-SHA, which is 0.1% higher than that of the base SGN. In this experiment, the accuracy of each detailed action class between the base SGN and the proposed SGN-SHA was compared using a confusion matrix, as shown in Figure 3. Although both methods showed a similar accuracy for detailed action classes, they achieved the lowest accuracy in the ‘pick up with one hand’ class, with SGN at 75% and SGN-SHA at 83%. In the case of the SGN, the accuracy was 100% in three classes: ‘pick up with two hands’, ‘drop trash’, and ‘donning’. By contrast, SGN-SHA showed 100% accuracy in the three classes of ‘pick up with two hands’, ‘stand up’, and ‘donning’, and thus the results of the two methods were slightly different. However, overall, there were no significant differences in the accuracy of each action class between the two methods, and thus we can see that SGN-SHA has a similar performance as the base model despite having fewer parameters and a lower computational complexity.

## 5. Conclusions

In this study, we proposed a new type of SGN-SHA approach that transforms the GCN of the base SGN into SHA to improve the action recognition. To solve the problem of a GCN, the core of an SGN, which expresses global features better than local features owing to its structural characteristics, we propose an SHA spatial feature expression method that can simultaneously consider the global and local features of the joints. Using SHA, it is possible to significantly reduce the number of parameters and computational complexity of the SGN. Based on the experimental results, it was confirmed that the performance was maintained despite a 50% reduction in the number of model parameters and a 2.6-fold reduction in the computational complexity. In terms of the amount of computation, real-time operation is possible in low-end devices.

As such, the advantage of a lightweight network using SGN-SHA is not only the promising action recognition accuracy rate, but also faster speed and fewer operations. The proposed action recognition algorithm can be deployed to robots, surveillance cameras, AR, VR and other embedded devices where real-time recognition is important.

In future research, we plan to expand this study to the development of a real-time action recognition model that can reduce the computational complexity while improving the action recognition performance by fusing skeleton sequences and RGB video frames.

## Figures and Tables

**Table 1:** Accuracy, the numbers of parameters (Params, M: Million) and computation complexity (Giga FLoating point Operation Per Second: GFLOPs) between SoTA methods and the proposed approach on the NTU60 dataset.

Method	NTU60		Lightweight	
	XSub (%)	XView (%)	Params (M)	GFLOPs
SGN [1]	89.0	94.5	0.69	0.16
ST-GCN [4]	81.5	88.3	3.10	16.3
Shift-GCN [12]	90.7	96.5	0.76	10.0
Info-GCN [25]	93.0	97.1	1.56	1.83
MS-G3D [26]	91.5	96.2	3.50	48.8
DSTA-Net [27]	91.5	96.4	3.40	55.6
3s-AimCLR [28]	86.9	92.8	2.51	1.86
RA-GCNv2 [29]	87.3	93.6	6.21	32.80
SGN-SHA	87.5	92.6	0.32	0.062

**Table 2:** Accuracy, numbers of parameters, and computation complexity between SoTA methods and the proposed method on the NTU120 dataset.

Method	NTU120		Lightweight	
	XSub (%)	XSet (%)	Params (M)	GFLOPs
SGN [1]	79.2	81.5	0.69	0.16
MS-G3D [4]	86.9	88.4	3.50	48.8
Shift-GCN [12]	85.9	87.6	0.76	10.0
Info-GCN [25]	89.8	91.2	1.56	1.83
DSTA-Net [27]	86.6	89.0	3.40	55.6
3s-AimCLR [28]	80.1	80.9	2.51	1.86
RA-GCNv2 [29]	81.1	82.7	6.21	32.80
SGN-SHA	78.5	87.1	0.32	0.062

**Table 3:** Differences in accuracy, number of parameters, and computational complexity according to the number of attention heads in the NTU60-XView dataset.

Head Number	NTU60-XView		
	Accuracy (%)	Params (M)	GFLOPs
1	92.6	0.32	0.062
2	94.3	0.95	0.29
3	94.3	2.36	0.77
4	94.4	4.65	1.62
5	94.5	8.03	2.92

## Data Availability

Not applicable.
